# Evaluation of WHO immunologic criteria for treatment failure: implications for detection of virologic failure, evolution of drug resistance and choice of second-line therapy in India

**DOI:** 10.7448/IAS.16.1.18449

**Published:** 2013-06-03

**Authors:** Snigdha Vallabhaneni, Sara Chandy, Elsa Heylen, Maria L Ekstrand

**Affiliations:** 1 Center for AIDS Prevention Studies and the Division of Infectious Diseases, University of California, San Francisco, CA, USA; 2 St John's National Academy of Health Sciences, Bangalore, India; 3 Center for AIDS Prevention Studies, University of California, San Francisco, CA, USA; 4 St John's Research Institute, Bangalore, India

**Keywords:** virologic failure, immunologic failure, WHO criteria, genotype, resistance, India, resource-limited settings

## Abstract

**Introduction:**

Routine HIV viral load (VL) testing is not available in India. We compared test performance characteristics of immunologic failure (IF) against the gold standard of virologic failure (VF), examined evolution of drug resistance among those who stayed on a failing regimen because they did not meet criteria for IF and assessed implications for second-line therapy.

**Methods:**

Participants on first-line highly active antiretroviral therapy (HAART) in Bangalore, India, were monitored for 24 months at six-month intervals, with CD4 count, VL and genotype, if VL>1000 copies/ml. Standard WHO criteria were used to define IF; VF was defined as having two consecutive VL>1000 copies/ml or one VL>10,000 copies/ml. Resistance was assessed using standard International AIDS Society-USA (IAS-USA) recommendations.

**Results:**

Of 522 participants (67.6% male, mean age of 37.5; 85.1% on nevirapine-based and 40.4% on d4T-containing regimens), 57 (10.9%) had VF, 38 (7.3%) had IF and 13 (2.5%) had both VF and IF. The sensitivity of immunologic criteria to detect VF was 22.8%, specificity was 94.6% and positive predictive value was 34.2%. Forty-four participants with VF only continued on their failing first-line regimen; by the end of the study period, 90.9% had M184V, 63.6% had thymidine analogue mutations (TAMs), 34.1% had resistance to tenofovir, and 63.6% had resistance to etravirine.

**Conclusions:**

WHO IF criteria have low sensitivity for detecting VF, and the presence of IF poorly predicts VF. Relying on CD4 counts leads to unnecessary switches to second-line HAART and continuation of failing regimens, jeopardizing future therapeutic options. Universal access to VL monitoring would avoid costly switches to second-line HAART and preserve future treatment options.

## Introduction

An estimated 6.6 million HIV-infected individuals are receiving highly active antiretroviral therapy (HAART) in resource-limited settings [1]. In India alone, approximately 400,000 individuals have been started on HAART between 2004 and 2011 [2]. Although this rapid scale-up of HAART has led to dramatic declines in mortality, many challenges remain, including optimal monitoring for treatment failure, the threat of emerging drug resistance and the availability of second-line therapy.

Treatment failure can be defined as progression of disease after initiation of HAART. Failure can be assessed by clinical (the appearance of new opportunistic infections, on-going weight loss, etc.), immunologic (a decline in CD4 count) or virologic (a viral rebound above a set threshold of 200 copies/ml) criteria [3]. It has been well established that virologic failure (VF) precedes immunologic failure (IF), which in turn precedes clinical failure [4]. A detectable viral load (VL) is among the earliest signs of treatment failure, and continuing a failing regimen has been associated with increased morbidity and mortality [5,6] as well as accumulation of drug resistance mutations that further limit second-line options [7]. Therefore, routine virologic monitoring is the standard of care in the developed world [8]. However, because of cost and limited access to laboratory technology, routine VL monitoring is not performed or recommended in resource-limited settings [9]. Instead, clinicians in these settings rely on defining treatment failure based on CD4 cell counts, clinical manifestations of disease progression, or both. Several studies from resource-limited settings have shown that lack of VL monitoring leads to frequent misclassification of failure and therefore either premature switching to second-line regimens or a delay in change to second-line agents [10–17].

The aims of this longitudinal analysis were to (1) evaluate the sensitivity, specificity and positive predictive value (PPV) of WHO-proposed criteria for treatment failure based on CD4 count, to detect true treatment failure based on virologic criteria in an Indian setting; and (2) assess the consequences of remaining on a failing regimen among those who do not meet criteria for IF but are, in fact, failing by virologic criteria, by examining the evolution of drug resistance and implications for second-line antiretroviral therapy.

## Methods

The data presented in the present study were collected as part of a two-year observational cohort study of adherence to HAART among HIV-infected adults attending public and private clinics in Bangalore, India. The full methods of this study have been published elsewhere [18].

### Setting

The study was conducted at three clinics (one public, one private, and one public-private partnership clinic) in Bangalore, India. Bangalore is a major metropolitan city in Karnataka, which is one of the six states in India with a high HIV prevalence [2]. The most commonly prescribed antiretroviral therapy in this setting is a generic nevirapine-based regimen, usually including lamivudine and either zidovudine or stavudine (and, very infrequently, tenofovir). Patients with a concurrent diagnosis of active tuberculosis infection are placed on efavirenz-based regimens because of potential drug interactions with rifampicin. Patients attending the public and the public-private partnership clinic received their HIV medications free of cost [19].

Once patients are on a stable first-line regimen, they are monitored every six months, and treatment monitoring includes weight, haemoglobin, alanine aminotransferase (ALT), and CD4 count. Routine VL monitoring is not available. Patients suspected of having treatment failure for WHO-defined immunologic and clinical criteria are referred to select government HIV treatment centres designated as Centers for Excellence for evaluation for second-line therapy [20]. Beginning in 2011, recommendations of the National AIDS Control Organization (NACO) of India were to obtain targeted VL testing for those with suspected treatment failure to avoid unnecessary switches to second-line therapy. The recommended second-line regimen is a combination of zidovudine or tenofovir, lamivudine, and ritonavir-boosted atazanavir.

### Participants

Participants were eligible if they were 18 years or older, were HIV infected, were on antiretroviral therapy for at least one month (and therefore not HAART naïve) at the time of enrolment, and were willing to participate in all follow-up visits. A total of 533 consecutive patients seeking care at the three clinic sites in Bangalore who met eligibility criteria were enrolled and followed in the study between August 2007 and December 2011. In this analysis, we included the 522 patients who had CD4 and VL data available at time points six or more months after starting HAART.

### Procedures

The study was approved by the institutional review boards of the University of California–San Francisco and St John's National Academy of Health Sciences.

Potential participants were referred to the study interviewers by clinic staff. Following referral, participants were taken to a separate room for eligibility confirmation and the study interview. If found eligible, participants were monitored every 3 months for 24 months and were interviewed to assess adherence. Blood work was done every six months. All patients provided written informed consent.

Participants’ blood was drawn by trained staff phlebotomists. CD4 cell count, HIV plasma VL, HIV genotype, and if VL>1000 copies/ml were assessed at six-month intervals. CD4 cell counts were performed on whole-blood specimens collected in an EDTA (ethylenediaminetetraacetic acid) tube using a single-platform flow cytometry assay (PCA system; Guava Technologies Inc., Hayward, CA, USA). A real-time polymerase chain reaction (PCR) assay with a fluorescein-labelled TaqMan probe was used for the quantitation of VL. The test was developed and its performance characteristics were determined at Molecular Diagnostics and Genetics, Reliance Life Sciences (Mumbai, India). This assay can reliably detect an HIV RNA level of 100 copies/ml of blood. The HIV-1 drug resistance assay was performed at YRGCARE Infectious Diseases Laboratory (Chennai, India) using an in-house method [21]. The performance characteristics of this method have been validated [22], and the assay is certified by the TREAT Asia Quality Assurance (TAQAS) Program (National Serology Reference Laboratory, Australia). Of note, we did not have baseline genotypes on these participants. Furthermore, we did not assess for the presence of protease inhibitor (PI)-associated mutations because PIs were very rarely used in the setting at the time of the study.

The results of the VL testing were provided to the patients and their treating clinicians. Providers then were able to use these results as well as other relevant information to make treatment-related decisions for each patient, such as clinical data; national guidelines at the time the study was conducted; and, for patients in the private clinic setting, their ability to pay for second-line therapy. These physicians may have been limited in their ability to refer to second-line therapy because second-line therapy was available only through small pilot programmes in a few centrally located care settings in India.

### Definitions

We used standard WHO-defined criteria for IF in this analysis since these are the same criteria adopted by NACO in India. A participant was categorized as having IF if he or she met one of the following three criteria after at least six months on HAART: (1) fall of CD4 count to pre-therapy baseline or below, (2) ≥50% fall of absolute CD4 count from the on-treatment peak value or (3) persistent CD4 levels below 100 cells/mm^3^.

VF was defined as, after at least six months on HAART, having a VL greater than 1000 copies/ml at two consecutive measurements (six months apart at study visits) or one VL measure >10,000 copies/ml.

We used the International AIDS Society-USA (IAS-USA) recommendations [23] to categorize genotypic mutations. Nucleoside reverse transcriptase inhibitor (NRTI) mutations included in this analysis were as follows: M184V, M184I and M184V/I for lamivudine (3TC) and emtricitabine (FTC) resistance; K65R and K70E, associated with tenofovir (TDF) resistance; thymidine analogue mutations (TAMs) M41L, D67N, K70R, L210W, T215Y, T215F, K219Q, and K219 E, associated with resistance to multiple NRTIs; and multinucleoside mutations, including the 69 insertion complex and 151 complex. Tenofovir resistance was defined as the presence of three or more TAMs, two of which included 41, 210, and 215 mutations; or the presence of K65R or 69 insertion site mutations. Non-nucleoside reverse transcriptase inhibitor (NNRTI) mutations that we assessed included K103N, Y181C, Y181I, G190A, G190S, V108I, Y188L, V106M, K103NS, K101E and G190E. Etravirine mutation scores were calculated using the scoring system described by Vingerhoets et al. [24] and recommended by the IAS-USA.

### Data analysis

Descriptive statistics includes means, standard deviations, medians and proportions when appropriate. Performance of the immunologic criteria was assessed by calculating their sensitivity, specificity and PPV, with exact confidence intervals, using VF as the “gold standard.” These analyses were performed both on the sample as a whole, and for males and females separately. One participant who identified as transgender was included in the analyses of the whole sample, but excluded from the analyses by gender. Differences between men and women were assessed via a *t*-test (age), Mann-Whitney *U*-test (CD4 and time on HAART), or *χ* ^2^-test (categorical variables). Due to small numbers, no gender-specific analyses were done on the genotyping data. Analyses were done in SPSS, version 18.0.2 (SPSS Inc., Chicago, IL, USA), and Stata, version 12.1 (StataCorp LP, College Station, TX, USA).

## Results

Five-hundred and twenty-two participants were followed for a median of 24 months. Two-thirds of participants were male (*n*=353; 67.6%), and the mean age was 37.5 (standard deviation (SD): 8.5) (Table 1). At the time of study enrolment, participants had been on HAART for a median of 17 months (interquartile range (IQR): 6–30 months) and median CD4 at that time was 333 (IQR: 210–470). A vast majority of participants (*n*=444, 85.1%) were on nevirapine-based regimens, and 40.4% (*n*=211) were on d4T-containing regimens. Seventy-seven per cent of participants were on their first-ever ART regimen; the remaining were also on first-line agents, but may have undergone some treatment modification for toxicity or drug interactions. Female patients were significantly younger (mean age: 34.3 years for females compared to 39.0 years for males) and had a higher median CD4 count at study enrolment (362 for females versus 315 for males) (Table 1).

**Table 1:** Demographic and clinical characteristics at study enrolment of 522 patients on HAART, Bangalore, India (2007–2011): *n* (%)

Variables	All (*n*=522)	Men (*n*=353)	Women (*n*=168)	*p* a
Gender				
Male	353 (67.6)			
Female	168 (32.2)			
Transgender^b^	1 (0.2)			
Patient age: mean (SD)	37.5 (8.5)	39.0 (8.2)	34.3 (8.2)	<0.001
Care setting				0.005
Public	279 (53.5)	198 (56.1)	81 (48.2)	
Private	91 (17.4)	68 (19.3)	23 (13.7)	
Public-private	152 (29.1)	87 (24.6)	64 (38.1)	
HAART regimen				0.106
3TC +D4T+NVP	180 (34.5)	114 (32.3)	65 (38.7)	
3TC+AZT+NVP	261 (50.0)	180 (51.0)	81 (48.2)	
Other NVP based	3 (0.6)	1 (0.3)	2 (1.2)	
3TC+D4T+EFV	31 (5.9)	20 (5.7)	11 (6.5)	
3TC+AZT+EFV	40 (7.7)	33 (9.3)	7 (4.2)	
TDF+FTC+EFV	6 (1.1)	5 (1.4)	1 (0.6)	
Protease inhibitors	1 (0.2)	0 (0.0)	1 (0.6)	
Median (IQR) CD4 cells/µl	333 (210–470)	315 (195–434)	362 (243–509)	0.004
Median (IQR) duration on HAART, in months	17 (6–30)	18 (7–30)	16 (4–31)	0.433

SD, standard deviation; IQR, interquartile range.

a Based on *t*-test for age, exact *χ* ^2^-test for care setting and regimen, and Mann-Whitney *U*-test for CD4 and duration on HAART.

b Excluded from analyses by gender.

A total of 57 (10.9%) patients experienced VF during the study; 13 of the 57 patients met criteria for IF at the time VF was detected, leaving 44 patients with VF only (Table 2). An additional 25 patients (4.8%) met criteria only for IF based on one or more of three WHO-defined criteria for IF. The sensitivity of the WHO IF criteria to detect VF was therefore 22.8% (95% confidence interval (CI): 12.7%–35.8%), the specificity was 94.6% (95% CI: 92.2%–96.5%), and the PPV was 34.2% (95% CI: 19.6%–51.4%). There were no differences in the occurrence of VF by gender (40/353 men and 17/168 women). Sensitivity, specificity and PPV also did not differ by gender.

**Table 2:** Test characteristics of WHO immunologic failure criteria to detect virologic failure

	VF+	VF−	Total
IF+	13	25	38
IF−	44	440	484
Total	57	465	522

Positive predictive value: 34.2% (95% CI: 19.6–51.4%); sensitivity: 22.8% (95% CI: 12.7–35.8%); specificity: 94.6% (95% CI: 92.2–96.5%).

Of the 44 total patients with VF only, 19 (43.2%) eventually developed IF during the study follow-up period. Median time from VF to IF in this subset of patients was 18 months. We assessed the evolution of drug resistance in these 44 patients between the time VF was detected and either the end of the study period or the time when IF was detected, when presumably the patient would be referred for second-line therapy. Twenty-nine of these 44 patients already had VF at enrolment into the study and had been on therapy for a median of 25 months at the time of their initial study visit (Table 3). A majority (86.2%; *n*=25) of participants with VF at their baseline visit had an M184V mutation. Nearly one-half (48.3%; *n*=14) of these participants had at least one TAM: 20.7% (*n*=6) had one TAM, 17.2% (*n*=5) had two TAMs, and 10.3% (*n*=3) had three or more TAMs, indicating that they may have been failing for many months by the time they enrolled in the study. Additionally, two participants were found to have the 69 insertion site mutation, and four more patients had K65R or K70E. Six of the 29 participants (20.7%) had evidence of genotypic resistance to tenofovir. With regard to NNRTI mutations, more than one-half of the participants with VF at enrolment (51.7%; *n*=15) had Y181C owing to the prevalence of nevirapine use in first-line regimens. Seven (24.1%) had K103N, seven (24.1%) had G190A and six (20.7%) had K101E. All but two patients (93.1%) were resistant to both nevirapine and efavirenz. Based on the NNRTI mutations that were present, and using the weighted genotypic score for the determination of etravirine susceptibility as outlined above, 18 of the 29 participants (62.1%) had a calculated etravirine score of 2.5 or greater, corresponding to intermediate- or high-level resistance to etravirine.

**Table 3:** Consequences of staying on a failing regimen on viral genotype, at study enrolment, during the study, and at the end of the study (*N*=44)

	At time VF detected^a^			
			
Mutation	VF at enrolment (*N*=29)	Incident VF during study (*N*=15)	During study^b^ (*N*=44)	End of study^c^ (*N*=44)
NRTI mutations				
M184V/I	25 (86.2%)	8 (53.3%)	+7	40 (90.9%)
Any TAMS	14 (48.3%)	0	+20	28 (63.6%)
1 TAM	6	0		8
2 TAMs	5	0		11
≥3 TAMS	3	0		9
69/Q151M	2 (6.9%)	0	+5	7 (15.9%)
K65R/70E	4 (13.8%)	0	+0	4 (9.1%)
TDF resistance	6 (20.7%)	0	+9	15 (34.1%)
NNRTI mutations				
K103N	7 (24.1%)	2 (13.3%)	+3	12 (27.3%)
Y181C	15 (51.7%)	2 (13.3%)	+2	19 (43.2%)
G190A	7 (24.1%)	5 (33.3%)	+6	18 (40.9%)
K101E	6 (20.7%)	1 (6.7%)	+6	13 (29.5%)
ETR score>2.5	18 (62.1%)	2 (13.3%)	+8	28 (63.6%)

a “At time VF detected” refers to mutations present in two sets of participants. Twenty-nine of the 44 participants who met criteria for VF only and no IF already had virologic failure at the time of enrolment in the study, when the first viral load was measured. Median time on therapy at enrolment for these participants was 25 months (VF at enrolment). Fifteen additional participants developed VF during the study period, meaning the first viral load measured during the study was suppressed but was detectable at a subsequent time point in the study (incident VF).

b “During study column” refers to additional mutations accumulated during the study period among all 44 patients who met criteria for VF but not IF during the study.

c “End of study column” refers to the presence of particular mutations on the last available genotype for all 44 patients who met criteria for VF but not IF during the study.

Fifteen participants developed VF during the study. The only NRTI mutation detected in any of these participants was M184V, with eight of the 15 patients (53.3%) harbouring this mutation. None of the participants who developed VF during the study were resistant to tenofovir at the time that treatment failure was detected. There were a few NNRTI mutations present: two patients had Y181C, two had K103N, five had G190A and one had K101E. A total of nine (60.0%) were resistant to nevirapine and efavirenz. However, only two patients (13.3%) had an etravirine mutation score of ≥2.5.

The 44 participants with VF only and without evidence of IF (includes 29 with VF at baseline and the 15 who developed VF during the study) were followed for a median of 18 months (either until the end of the study or until they developed evidence of IF) while they remained on a failing regimen. During this time, seven additional participants developed M184V, three developed 69 insertion site mutations and two developed Q151M. Twenty additional TAMs also developed in the 44 participants. When the evolution of NNRTI mutations was assessed, three more participants developed K103N, two more developed Y181C, and six more each developed G190A and K101E. By the end of the study period, 34.1% of participants (*n*=15) were found to have mutations that would confer high-level resistance to tenofovir. Based on the NNRTI mutations present, 63.6% (*n*=28) of participants had etravirine mutation scores ≥2.5, associated with intermediate- or high-level resistance to etravirine.

Mutation patterns among the 13 participants with VF and IF detected at the same time were also examined and found to be comparable to the mutation patterns at the end of the study for the 44 participants with VF but no IF: 9 of the 13 participants (69.2%) had the M184V mutation; six (46.2%) had any TAMs, with a majority having 3 or more TAMs; 8 (61.5%) had predicted resistance to tenofovir; and 9 (69.2%) had an etravirine mutation score ≥2.5.

## Discussion

In this longitudinal study of 522 HIV-infected participants on first-line HAART in Bangalore, India, we found that WHO-defined criteria for IF are insensitive at detecting virologic treatment failure and, more importantly, that the delay in diagnosis of treatment failure results in accumulation of a substantial number of drug-resistant mutations and has the potential to have a significant impact on the efficacy of second-line antiretroviral treatment.

Participants who developed VF during the study period, and thus were recognized as having treatment failure immediately, did not have any mutations associated with tenofovir resistance at the time of VF. However, by the end of a follow-up period of a median of 18 months, when participants finally developed evidence of IF, one in three participants had mutations that conferred high-level resistance to tenofovir. Subtype C HIV-1 virus is predominant in India, and it has been previously established that in this subtype, K65R is selected for by on-going exposure to d4T and leads to tenofovir cross-resistance [25]. Although NACO is now moving towards phasing out the use of d4T, nearly half of the participants in our study were on d4T-containing regimens. On-going exposure to thymidine NRTIs, including d4T and azidothymidine (AZT), in the setting of on-going viral replication contributed to further accumulation of K65R and TAMs, resulting in substantial levels of resistance to tenofovir. Although tenofovir resistance was not specifically addressed, studies from northern India and Kenya among patients failing first-line HAART also found similarly high rates of both NRTI and NNRTI resistance [26,27].

Furthermore, based on the presence of TAMs and other NRTI mutations, a substantial proportion of individuals in our cohort also had complete resistance to AZT and d4T, essentially making the whole NRTI class of drugs a nonviable option for a significant number of patients needing second-line therapy. Similar concerns have been reported in studies from Africa, where 17%–53% of patients with treatment failure had no active NRTI options [28–30]. This is especially alarming given the extremely limited access to second-line regimens. Salvaging patients with complete NRTI resistance could potentially require the use of newer agents such as raltegravir, maraviroc, etravirine and darunavir in combination with one another [31,32]; however, none of these drugs are widely available in these settings.

According to India's 2011 national guidelines, when patients meet the criteria for IF, they are tested to confirm VF and then empirically started on ritonavir-boosted atazanavir, tenofovir and lamivudine. In nearly one-third of patients, this would mean that they will be receiving PI monotherapy since the other two drugs in the regimen are completely inactive or only partially active. Although a recent AIDS Clinical Trials Group (ACTG) study has shown some promise with lopinavir-ritonavir monotherapy in resource-limited settings in the short term [33], this may not a viable long-term option. One report from India on patients with PI-based second-line therapy after first-line therapy failure showed that 72% were still viremic and a substantial proportion developed PI mutations while on PI-based second-line regimens [34]. Nearly one-quarter developed mutations associated with darunavir in this study. Therefore, preserving NRTI options with early detection of VF is imperative to preserve treatment success in resource-limited settings.

Etravirine is a second-generation NNRTI that has been shown to be effective in achieving virologic suppression when given in combination with other fully active drugs in patients with prior treatment experience [35]. However, treatment with first-generation NNRTIs such as nevirapine, especially if there is on-going replication in the setting of VF, may lead to cross-resistance to etravirine. In participants with incident VF (VF detected after the baseline study visit), only 13% had the predicted resistance to etravirine at the time that failure was detected. This is in contrast to the 64% with predicted resistance by the end of the study, when participants also developed criteria for IF. High prevalence of resistance to etravirine among patients failing first-line HAART has previously been reported in India [36]. Therefore, when routine VL monitoring is not available and those with IF have likely been failing for a substantial period, they are expected to have accumulated enough NNRTI mutations to make etravirine a nonviable option. Although etravirine is not being considered by WHO or NACO as part of a second-line regimen, these findings confirm that in a setting where first-line regimens are almost exclusively NNRTI-based regimens and second-line regimens are chosen empirically, etravirine would not be an effective agent.

Our findings regarding the sensitivity of WHO's IF criteria to detect VF are in line with similar studies conducted in Africa, where subtype C virus also predominates. Sensitivity of WHO-defined criteria for IF to detect VF ranged from 21 to 58% [10–14], depending on the setting in which the study was conducted and the definition used for VF. We believe that the sensitivity we report in this study is on the lower end of the reported range because of its longitudinal nature. Most of the other studies were conducted cross-sectionally, allowing for the possibility that VF had been occurring for a longer duration and hence participants have enough time for IF to develop as well. In our study, we had a mixture of participants, some of whom may have been failing for some time at enrolment, and an additional 15 patients who developed VF during the study period. Since we know that IF develops after VF, we would not expect these 15 participants to have had a chance to develop IF at the time they were initially determined to have treatment failure.

The prevalence of VF was fairly low in our setting: approximately 10% of participants were found to have VF. This contributed to the low PPV of 34%. However, this number is slightly lower than what others have reported: 36.8% in South Africa [10], 39% in a large study in Nigeria [13] and 70% in a government programme in India [12]. If IF criteria were used to define treatment failure, a substantial number of patients would be unnecessarily switched to more expensive second-line therapy. This adds not only cost to the national programme but also risks for developing resistance to second-line agents for those who do not have access to third-line agents. Fortunately, starting in 2010, WHO has started recommending targeted VL testing for those suspected of having IF to avoid switching patients with suppressed VLs to second-line therapy. NACO also endorsed these guidelines in 2011.

There are several limitations to this study. Patients were not HAART naïve at the time of enrolment, and we did not have baseline genotypes to determine the contribution of transmitted drug resistance to, or the impact of previous treatment regimens on, virologic treatment failure. However, studies from other parts of India show that the prevalence of drug resistance in HAART-naïve patients is between 3% and 5% in south India [37–39]. Second, because patients were enrolled into the study at any point one or more months after initiating HAART and the median time on treatment at study enrolment was 17 months, some participants had VF that may have been on-going for many months by the time the first VL was obtained. Because of this limitation, we were not able to calculate the rate of accumulation of mutations, since it would not be the same for those who had been failing for some time and those who developed VF during the study. Finally, we did not look for PI resistance when we conducted our genotypes because of the cost associated with performing PI resistance analysis. However, given that exposure to PIs is extremely low, and none of our patients were on PI-based first-line regimens, we do not think that this is a significant drawback of the study.

## Conclusions

In resource-limited settings, HIV drug resistance is a threat to the long-term effectiveness of antiretroviral treatment. It is unlikely that resistance testing will be available at the level of the individual patient in the near future. Additionally, treatment changes based on individual patient testing are not feasible due to limited access to second- and third-line agents. The best way to ensure treatment success with second-line agents may be through early detection of treatment failure. WHO IF criteria have low sensitivity for detecting VF, and the presence of IF poorly predicts VF. Relying on CD4 count data alone would lead to a substantial number of unnecessary switches to second-line HAART. Although there are some conflicting studies [40], routine VL monitoring has been shown to be cost saving in resource-limited settings, especially when unnecessary treatment switches are taken into account [41]. A notable proportion of patients would be continued on first-line therapy that they are already failing, allowing for accumulation of drug resistance and, possibly, subsequent transmission of resistance to new partners [42,43]. Universal access to VL monitoring has the potential to have enormous public health impact because it would help avoid costly switches to second-line HAART, limit the development of drug resistance and transmission, and preserve future therapeutic options.

## References

[1] Joint United Nations Programme on HIV/AIDS (UNAIDS) (2011). AIDS at 30: nations at the crossroads. http://www.unaids.org/unaids_resources/aidsat30/aids-at-30.pdf.

[2] National AIDS Control Organization Patients alive and on ART August 2010. http://nacoonline.org/upload/Care%20&%20Treatment/Patients%20alive%20and%20on%20ART/People%20alive%20and%20on%20ART%20August%202010.pdf.

[3] Aldous JL, Haubrich RH (2009). Defining treatment failure in resource-rich settings. Curr Opin HIV AIDS.

[4] Deeks SG, Barbour JD, Martin JN, Swanson MS, Grant RM (2000). Sustained CD4+T cell response after virologic failure of protease inhibitor-based regimens in patients with human immunodeficiency virus infection. J Infect Dis.

[5] Haubrich RH, Currier JS, Forthall DN, Beall G, Kemper CA, Johnson D (2001). A randomized study of the utility of human immunodeficiency virus RNA measurement for the management of antiretroviral therapy. Clin Infect Dis.

[6] Petersen ML, van der Laan MJ, Sonia N, Eron JJ, Moore RG, Deeks SG (2008). Long-term consequences of the delay between virologic failure of highly active antiretroviral therapy and regimen modification. AIDS.

[7] Hatano H, Hunt P, Weidler P, Coakley E, Hoh R, Liegler T (2006). Rate of viral evolution and risk of losing future drug options in heavily pretreated, HIV-infected patients who continue to receive a stable, partially suppressive regimen. Clin Infect Dis.

[8] Panel on Antiretroviral Guidelines for Adults and Adolescents Guidelines for the use of antiretroviral agents in HIV-1-infected adults and adolescents. http://www.aidsinfo.nih.gov/contentfiles/lvguidelines/adultandadolescentgl.pdf.

[9] World Health Organization (2010). Antiretroviral therapy for HIV infection in adults and adolescents. Recommendations for a public health approach [2010 revision]. http://whqlibdoc.who.int/publications/2010/9789241599764_eng.pdf.

[10] Mee P, Fielding KL, Charalambous S, Churchyard GJ, Grant AD (2008). Evaluation of the WHO criteria for antiretroviral treatment failure among adults in South Africa. AIDS.

[11] Kantor R, Diero L, Delong A, Kamle L, Muyonga S, Mambo F (2009). Misclassification of first-line antiretroviral treatment failure based on immunological monitoring of HIV infection in resource-limited settings. Clin Infect Dis.

[12] Rewari BB, Bachani D, Rajasekaran S, Deshpande A, Chan PL, Srikantiah P (2010). Evaluating patients for second-line antiretroviral therapy in India: the role of targeted viral load testing. J Acquir Immune Defic Syndr.

[13] Rawizza HE, Chaplin B, Meloni ST, Eisen G, Rao T, Sankalé JL (2011). Immunologic criteria are poor predictors of virologic outcome: implications for HIV treatment monitoring in resource-limited settings. Clin Infect Dis.

[14] Reynolds SJ, Nakigozi G, Newell K, Ndyanabo A, Galiwongo R, Boaz I (2009). Failure of immunologic criteria to appropriately identify antiretroviral treatment failure in Uganda. AIDS.

[15] Moore DM, Awor A, Downing R, Kaplan J, Montaner JS, Hancock J (2008). CD41 T-cell count monitoring does not accurately identify HIV-infected adults with virologic failure receiving antiretroviral therapy. J Acquir Immune Defic Syndr.

[16] van Oosterhout JJ, Brown L, Weigel R, Kumwenda JJ, Mzinganjira D, Saukila N (2009). Diagnosis of antiretroviral therapy failure in Malawi: poor performance of clinical and immunological WHO criteria. Trop Med Int Health.

[17] Moore DM, Mermin J, Awor A, Yip B, Hogg RS, Montaner JS (2006). Performance of immunologic responses in predicting viral load suppression: implications for monitoring patients in resource-limited settings. J Acquir Immune Defic Syndr.

[18] Ekstrand ML, Shet A, Chandy S, Singh G, Shamsundar R, Madhavan V (2011). Suboptimal adherence associated with virological failure and resistance mutations to first-line highly active antiretroviral therapy (HAART) in Bangalore, India. Int Health.

[19] Shet A, DeCosta A, Heylen E, Shastri S, Chandy S, Ekstrand M (2011). High rates of adherence and treatment success in a public and public-private HIV clinic in India: potential benefits of standardized national care delivery systems. BMC Health Serv Res.

[20] National AIDS Control Organization Antiretroviral therapy guidelines for HIV infected adults and adolescents including post-exposure 2007. http://nacoonline.org/upload/Policies%20&%20Guidelines/1.%20Antiretroviral%20Therapy%20Guidelines%20for%20HIV-Infected%20Adults%20and%20Adolescents%20Including%20Post-exposure.pdf.

[21] Balakrishnan P, Kumarasamy N, Kantor R, Solomon S, Vidya S, Mayer KH (2005). HIV type 1 genotypic variation in an antiretroviral treatment-naive population in southern India. AIDS Res Hum Retroviruses.

[22] Saravanan S, Vidya M, Balakrishnan P, Kumarasamy N, Solomon SS, Solomon S (2009). Evaluation of two human immunodeficiency virus-1 genotyping systems: ViroSeq 2.0 and an in-house method. J Virol Methods.

[23] Johnson VA, Calvez V, Günthard HF, Paredes R, Pillay D, Shafer RW (2011). Update of the drug resistance mutations in HIV-1. Top Antivir Med.

[24] Vingerhoets J, Tambuyzer L, Azijn H, Hoogstoel A, Nijs S, Peeters M (2010). Resistance profile of etravirine: combined analysis of baseline genotypic and phenotypic data from the randomized, controlled phase III clinical studies. AIDS.

[25] Deshpande A, Jeannot AC, Schrive MH, Wittkop L, Pinson P (2010). Analysis of RT sequences of subtype C HIV-type 1 isolates from Indian patients at failure of a first-line treatment according to clinical and/or immunological WHO guidelines. AIDS Res Hum Retroviruses.

[26] Sinha S, Shekhar RC, Ahmad H, Kumar N, Samantaray JC, Sreenivas V (2012). Prevalence of HIV drug resistance mutation in the northern Indian population after failure of the first line antiretroviral therapy. Curr HIV Res.

[27] Arnedo M, Alonso E, Eisenberg N, Ibàñez L, Ferreyra L, Jaén A (2012). Monitoring HIV viral load in resource limited settings: still a matter of debate?. PLoS One.

[28] Wallis CL, Mellors JW, Venter WD, Sanne I, Stevens W (2010). Varied patterns of HIV-1 drug resistance on failing first-line antiretroviral therapy in South Africa. J Acquir Immune Defic Syndr.

[29] Hosseinipour MC, van Oosterhout JJ, Weigel R, Phiri S, Kamwendo D, Parkin N (2009). The public health approach to identify antiretroviral therapy failure: high-level nucleoside reverse transcriptase inhibitor resistance among Malawians failing first-line antiretroviral therapy. AIDS.

[30] Shepherd JCEM, Griffifth D, Charurat MEO High levels of classwide NRTI resistance among HIV-positive patients failing first-line antiretroviral regimens in Nigeria.

[31] Yazdanpanah Y, Fagard C, Descamps D, Taburet A, Collins C, Roquebert B (2009). High rate of virologic suppression with raltegravir plus etravirine and darunavir/ritonavir among treatment-experienced patients infected with multidrug-resistant HIV: results of the ANRS 139 TRIO trial. Clin Infect Dis.

[32] Lazzarin A, Campbell T, Clotet B, Johnson M, Katlama C, Moll A (2007). Efficacy and safety of TMC125 (etravirine) in treatment-experienced HIV-1-infected patients in DUET-2: 24-week results from a randomised, double-blind, placebo-controlled trial. Lancet.

[33] Bartlett JA, Ribaudo HJ, Wallis CL, Aga E, Katzenstein D, Stevens WS (2012). Lopinavir/ritonavir monotherapy after virologic failure of first-line antiretroviral therapy in resource-limited settings. AIDS.

[34] Saravanan S, Vidya M, Balakrishnan P, Kantor R, Solomon SS, Katzenstein D (2012). Viremia and HIV-1 drug resistance mutations among patients receiving second-line highly active antiretroviral therapy in Chennai, southern India. Clin Infect Dis.

[35] Ruxrungtham K, Pedro R, Latiff G, Conradie F, Domingo P, Lupo S (2008). Impact of reverse transcriptase resistance on the efficacy of TMC125 (etravirine) with two nucleoside reverse transcriptase inhibitors in protease inhibitor-naïve, nonnucleoside reverse transcriptase inhibitor-experienced patients: study TMC125-C227. HIV Med.

[36] Neogi U, Shet A, Shamsunder R, Ekstrand M (2011). Selection of nonnucleoside reverse transcriptase inhibitor-associated mutations in HIV-1 subtype C: evidence of etravirine cross-resistance. AIDS.

[37] Thorat SR, Chaturbhuj DN, Hingankar NK, Chandrasekhar V, Koppada R, Datkar SR (2011). Surveillance of transmitted HIV type 1 drug resistance among HIV type 1-positive women attending an antenatal clinic in Kakinada, India. AIDS Res Hum Retroviruses.

[38] Sinha S, Ahmed H, Shekahr RC, Kumar N, Samatarey JC, Sharma SK (2012). Prevalence of HIV drug resistance mutations in HIV type 1 isolates in antiretroviral therapy naïve population from northern India. AIDS Res Treat.

[39] Hingankar NK, Thorat SR, Deshpande A, Rajasekaran S, Chandrasekar C, Kumar S (2012). Initial virologic response and HIV drug resistance among HIV-infected individuals initiating first-line antiretroviral therapy at 2 clinics in Chennai and Mumbai, India. Clin Infect Dis.

[40] Kahn JG, Marseille E, Moore D, Bunnell R, Were W, Degerman R (2011). CD4 cell count and viral load monitoring in patients undergoing antiretroviral therapy in Uganda: cost effectiveness study. BMJ.

[41] Hamers RL, Sawyer AW, Tuohy M, Stevens WS, Rinke de Wit TF, Hill AM (2012). Cost-effectiveness of laboratory monitoring for management of HIV treatment in sub-Saharan Africa: a model-based analysis. AIDS.

[42] Phillips AN, Pillay D, Garnett G, Bennett D, Vitoria M, Cambiano V (2011). Effect on transmission of HIV-1 resistance of timing of implementation of viral load monitoring to determine switches from first to second-line antiretroviral regimens in resource-limited settings. AIDS.

[43] Gupta R, Jordan M, Sultan BJ, Hill A, Davis D, Gregson J (2013). Global trends in antiretroviral resistance in treatment-naive individuals with HIV after rollout of antiretroviral treatment in resource-limited settings: a global collaborative study and meta-regression analysis. Lancet.

